# Description and Clinical Management of Patients With Glanzmann's Thrombasthenia in a University Hospital, a Referral Center Specialized in Hemostasis, in Bogotá, Colombia

**DOI:** 10.7759/cureus.25657

**Published:** 2022-06-04

**Authors:** Maria H Solano, Karen Chaves, Claudia P Casas

**Affiliations:** 1 Hematology, San Jose Hospital – University Foundation of Health Sciences, Bogotá, COL

**Keywords:** glanzmann's thrombasthenia, viia factor, bleeding, platelet disorder, blood coagulation disorder

## Abstract

Introduction

Glanzmann's thrombasthenia (GT) is an autosomal recessive disorder of platelets caused by a deficiency in the glycoprotein IIb-IIIa. Bleeding from the skin, mucous membranes, and ecchymosis are symptoms manifested starting in early childhood. There may also be major bleeding conditions as a result of surgical procedures or trauma. The treatment is based on platelet transfusions, antifibrinolytic agents, and recombinant activated factor VII (rFVIIa).

Objective

To describe the demographic and clinical characteristics as well as the main treatment strategies used during bleeding events or procedures for patients diagnosed with GT that required inpatient attention at a university hospital, a referral center specialized in hemostasis, in the city of Bogota.

Materials and methods

A descriptive retrospective cohort study was done over a period of 10 years that included nine patients over 18 years of age diagnosed with GT.

Results

A total of 34 admissions were reported, 23 due to bleeding and 11 for scheduled surgery. Some of the admissions for bleeding (38%) (n=13) required surgical procedures. Overall, 23 surgical procedures were done, six of which were classified as major. Seventy-seven percent of the patients were women with a median age of 37. Their most common symptoms were mucosal and genitourinary bleeding. The use of antifibrinolytics was registered in 28 events, followed by the use of platelet transfusion in 19, and the use of rFVIIa in 17. The average hospital stay was eight days.

Conclusion

The characteristics registered and the treatments established for this cohort of Colombian patients with GT are similar to those reported in other hospitals around the world. GT presents diagnostic and therapeutic challenges and, therefore, acquiring more knowledge about this pathology is needed within this context.

## Introduction

Hereditary platelet disorders are severe and heterogeneous with a low prevalence in the population [1]. One of the main ones, Glanzmann's thrombasthenia (GT), is a disease with a reported low incidence. It was first documented in Bern in 1918 by the Swiss pediatrician Eduard Glanzmann who defined it as “hereditary hemorrhagic thrombasthenia” [2-3].

In 1964, Caen et al. [4] established the absence of platelet aggregation as the main diagnostic characteristic of GT. From that time on, platelet aggregation has been the most widely used diagnostic method although there are currently techniques such as flow cytometry, molecular, and genetic studies, which are not globally available.

In 1970, Nurden and Caen demonstrated that platelets in GT have an abnormality in the composition of membrane glycoproteins [5], where the integrin αIIbβ3 is partially expressed or its function has been affected when a mutation is introduced [2]. Recent reviews describe the wide range of mutations that produce this disease. Mutations occur across both genes, principally affecting ITGA2B, even though it is a smaller gene than ITGB3, it has 30 exons compared to 15 with double of splice sites. The platelets in GT can still adhere to the exposed subendothelium and start secreting from the granules. However, the subsequent reactions and thrombus formation are defective which leads to an aggregation failure in response to natural agonists, including ADP, collagen, and thrombin [6-7]. The symptoms are variable, the bleeding phenotype is multifactorial, and the majority of heterozygous patients are asymptomatic. In homozygous patients, the most frequent bleeding symptoms are purpura, epistaxis, gingival bleeding, and menorrhagia [2, 6, 8]. Gastrointestinal hemorrhage and hematuria are less frequent and are mostly associated with complications [6-8]. Muscle hematomas or hemarthrosis are very rare in this population [2, 6].

GT patients have a normal platelet morphology and, usually, a normal platelet count, prolonged bleeding time, altered platelet function assay (PFA), and a lack of or reduction in clot retraction [6]. Platelet aggregation in response to ADP, collagen, epinephrine, and thrombin is completely absent in GT [1-7]. When the abovementioned clinical and hemostasis diagnostic test findings are available, the deficiency of αIIbβ3 in platelets must be confirmed. There are techniques for this such as flow cytometry [6]. Mutations are mainly detected by the use of next-generation sequencing [3].

Treatment procedures are determined based on the area of bleeding and its severity. In general, local bleeding can be treated using local techniques such as fibrin sealants. Episodes of epistaxis and gingival bleeding can be controlled with nasal packing or topical foam applications for most patients [2]. In cases of severe bleeding, trauma or when surgery is necessary, platelet transfusion is usually required [6]. Likewise, different types of medications such as antifibrinolytics and rFVIIa have been used [9-10].

There are two reports in Colombia and Latin America that have been published in the literature that establish the procedures related to bleeding events or surgery [11-12]. Given the proposed scenario, the objective was to describe the demographic and clinical characteristics as well as the main treatment strategies used during bleeding events or procedures for patients diagnosed with GT at a university hospital, a referral center specialized in hemostasis, in the city of Bogota.

## Materials and methods

Study design

A descriptive retrospective cohort study included all patients diagnosed with GT treated in the hematology service unit at a university hospital over the 10-year period between 2009 and 2019. All patients over 18 years of age with a GT diagnosis and who required inpatient care were the criteria for inclusion. Information on the causes of hospitalization, type of bleeding, surgical procedure, and treatment received in each instance of hospitalization was collected.

Microsoft Excel® (Microsoft® Corp., Redmond, WA, USA) was used to build the database in which the demographic and clinical characteristics, as well as the clinical indications for hospitalization, surgical procedures, the various treatments received, complications, and mortality, were described. This study was endorsed by both the Research Committee of the School of Medicine and the Ethics Committee Fundación Universitaria de Ciencias de la Salud protocol number 1321-5068-36. The study was regarded as free of risks for the participants.

Analysis of data

The demographic and clinical characteristics of patients with GT, the causes of hospitalization, surgical procedures, treatment methods used, and outcomes were described. In some cases, a patient was hospitalized several times. The analysis of the results was done using the Stata statistical program version 12.0 (StataCorp LLC, College Station, TX, USA). The categorical variables were described in terms of absolute and relative frequencies and percentages using central tendency and dispersion measurements. The numerical variables were described as averages and standard deviations.

## Results

A total of 10 patients diagnosed with GT, treated in a university hospital, a referral center specialized in hemostasis, were studied over a period of 10 years. One patient was excluded because, after months of follow-up and treatment for GT, it was confirmed that his platelet disorder did not correspond to the GT pathology under study.

Description of the disease

The nine patients included were hospitalized on 34 occasions during this period of time (Table 1).

**Table 1:** Patient description F: Female, M: Male

Patient	Age at first hospital admission (years)	Sex	No. of hospital admissions
1	20	F	6
2	24	F	1
3	22	F	9
4	61	F	1
5	46	F	11
6	32	M	1
7	23	F	2
8	36	F	1
9	30	M	2

Of these, 23 episodes were secondary to bleeding and 11 events secondary to scheduled surgical procedures. However, some of the admissions due to bleeding required additional surgical procedures in 38% of the cases (n = 13). Overall, 23 surgical procedures were done, six of which were classified as major.

Most of the patients (77%) were female (n = 7). Their ages ranged between 20 and 61 years of age with a median of 37. The bleeding symptoms at the time of hospitalization were genitourinary in origin in 35% of the cases (n = 12). Of these, (n = 10) were due to abnormal uterine bleeding and (n = 2) to hematuria followed by mucosal bleeding in 26% of the cases (n = 9), and bleeding in the gastrointestinal tract in 21% (n = 7) (Table 2).

**Table 2:** Spectrum of clinical presentation at hospital admission GI: gastrointestinal bleeding

Cause of hospital admission	No. of patients	Percentage
Genitourinary bleeding	12	35
Mucosal bleeding	9	26
GI bleeding	7	21
Invasive procedures	5	15
Hemarthrosis and mucosal bleeding	1	3

The platelet counts were normal in all episodes of hospitalization. Most patients did not present any associated comorbidity. Only one patient had high blood pressure, and one had an infectious comorbidity. The latter patient had viral studies that documented infection with hepatitis C, who had a history of multiple transfusions.

Surgery

A total of 23 surgical procedures were carried out during the hospitalizations. These encompassed both scheduled and unscheduled surgery, of which 33% (n = 6) were major procedures including two childbirth deliveries, two genitourinary tract procedures (one hysterectomy and one myomectomy), two abdominal operations on the gastrointestinal tract (duodenectomy with multiple small bowel resections with end to side anastomosis). Only three of the major procedures required general anesthesia.

The total number of minor operations was 17, of which 41% were dental procedures such as endodontics, implants, and exodontia and 41% corresponded to upper endoscopic and/or colonoscopy procedures (n = 7), 12% ophthalmic procedures (n = 2), and 6% (n = 1) genitourinary tract procedures. Four patients required re-operation - two of them were upper gastrointestinal endoscopy and the other two dental procedures. Three of the re-operations were for mixed causes (platelet-related and mechanical) and one of them was secondary to a platelet-related origin.

Method of treatment

**Figure 1:**
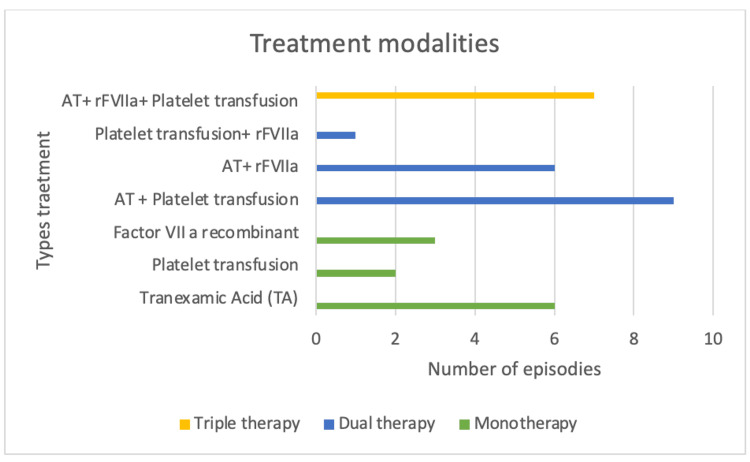
Types of treatment in hospital situations for patients with GT. Therapeutic options: -Monotherapy: A single treatment for hemostatic control. Tranexamic acid = TA; Platelet transfusion, recombinant Factor VIIa = rFVIIa. -Dual therapy: Two hemostatic therapeutic options in the same event. Includes the combinations: TA + platelets, TA + rFVIIa, Platelets + rFVIIa. -Triple therapy: Three hemostatic therapeutic options in the same event: TA + rFVIIa + platelets

The following were the treatment methods used in this study: antifibrinolytic monotherapy in six cases (18%), platelet transfusion in two (6%), and use of rFVIIa as monotherapy in three (9%). The combined treatment methods were platelet transfusion and antifibrinolytics in nine cases (26%), rFVIIa therapy and antifibrinolytics in six events (18%), and the use of platelet transfusion plus rFVIIa administration in one case (3%). Antifibrinolytics, platelet transfusion, and rFVIIa were used in conjunction in seven hospital admissions (20%) (Figure 1).

Of the total hospital admissions irrespective of treatment method, 19 patients received platelet transfusion, 17 patients received rFVIIa, and 28 patients received antifibrinolytics. For studies involving human subjects, appropriate approval must have been obtained from the relevant institutional review board or ethics committee. Before conducting the research, approval must have been obtained from an institutional review board (IRB) or ethics committee.

The administration of rFVIIa occurred between one and 23 days with an average days-of-use of five days and the administration of platelets was done between one and six days with an average of two days.

Hospitalization

The hospital stay ranged between one and 81 days, and four hospitalizations lasted 15 days or more. The duration of 60% of the hospitalizations was between one and five days. The average hospital stay was eight days for all cases. The case with the highest number of hospitalization days (81 days) was secondary to gastrointestinal tract bleeding and required major surgery (duodenectomy), ICU hospitalization, and surgical re-operation due to platelet-related and mechanical causes. Two cases required intensive care unit management, and in both this was related to the need for repeated surgery and difficulties with bleeding control. No mortality was reported during these hospitalizations.

## Discussion

GT is a low prevalent disease, however, it may be a common disease in populations with a high degree of consanguinity and in ethnic groups in Iran, India and Palestine [13]. It is an autosomal recessive disease produced by a defect in the function or in the quantity of glycoprotein IIb-IIIa (αIIb β3) of the platelet that is indispensable for platelet-fibrinogen bonding in endothelial lesions [2]. The genes that encode the glycoprotein are located in the long arm of chromosome 17 (q21-23 bands) in a 260-kb segment [8]. The ITGA2B gene encodes αIIb and the ITGB3 gene encodes β3. Mutations can occur in both genes, though ITGA2B is affected more than any other, possibly because of a higher probability of suffering single nucleotide substitutions that generate different types of mutations. To date, more than 80 mutations associated with this pathology have been identified [2, 14]. In GT, the platelets may adhere to the exposed subendothelium and initiate secretion from the granules. However, the subsequent reactions and thrombus formation are defective and, as a result, they fail to aggregate in response to natural agonists including ADP, collagen, and thrombin [7].

The disease is usually manifested starting in childhood with varied symptoms. The patients that are heterozygous are mostly asymptomatic. With homozygous patients, who may present anything from minimal bleeding to potentially fatal bleeding, the most commonly reported symptoms are: epistaxis, menorrhagia, gingival bleeding, hematuria, gastrointestinal bleeding, etc. [6, 8]. Normal platelet counts and morphology are found in the diagnosis. There are defects in platelet aggregation in response to all agonists. Ristocetin aggregation is usually normal. Integrin deficiency is confirmed by flow cytometry or monoclonal antibodies and, if possible, it is ideal to identify the specific associated mutation [6, 7]. In general, treatment can range from measures applied locally, the use of antifibrinolytics, to transfusion of platelets and use of rFVIIa depending on the type, location, and severity of the bleeding as well as the likely platelet intractability [9, 10, 15].

The data in the current study correspond to the observational study with the highest number of hospital admissions for patients diagnosed with GT in Colombia and Latin America. The clinical records of patients with GT were reviewed in order to describe the main clinical variables. Altogether, information was obtained from nine patients who accounted for 34 hospital admissions. The main cause of hospitalization was bleeding events, followed by the need for surgical procedures. The majority of these were women by 77% (n = 7). In literature, the male to female ratio was reported to be 1:1. The average age of the patients was 37 at the time of hospital admission, keeping in mind that this is an adult-only patient service. Eight of the patients were diagnosed prior to institutional admission. One of the patients was diagnosed at the institution through platelet aggregation [16-18].

The most frequent bleeding manifestations in the population were 35% genitourinary in origin, 30% mucosal bleeding, and 20% gastrointestinal tract bleeding, which is similar to what is reported in the literature [4, 19]. Including minor and major surgeries, there were 23 surgical procedures among the hospital cases. Surgical procedures are a challenge in the management of patients with GT because of the probability of bleeding, alloimmunization, and complications secondary to the surgery per se. In this study, just as reported in the literature, major surgery implied the use of general anesthesia, a greater number of days of hospitalization, and the possibility of complications as well as a greater number of days of platelet transfusion and the application of rFVIIa. Additionally, proper prevention and oral health care is very important for this group of patients in order to avoid dental procedures and emergencies [10, 20, 21].

Most bleeding episodes that require hospitalization or surgery are managed with systemic treatment. The use of antifibrinolytic drugs may be considered for monotherapy when the clinical manifestations are mild, and the need for other types of treatments is lower. Antifibrinolytics have the advantage of being cost-effective with an acceptable safety and tolerability profile. In non-randomized studies, a reduction in light-grade bleeding episodes has been demonstrated in patients with GT [9, 16, 19, 20]. In our cohort, the use of antifibrinolytic drugs as monotherapy was reported in six cases in which, the cause of hospital admission was mucous membrane bleeding or mild genital bleeding. Likewise, in combined treatments, tranexamic acid was used for the majority of the patients since it is generally used as initial therapy with the intention of using other associated systemic agents [9].

As a common procedure in the treatment of this type of patient, 60% of patients (n = 19) received platelet transfusion. In the study, there were reports of clinically evaluated platelet transfusion refractoriness in three of the patients and transfusion reactions requiring pre-medication in 26% (n = 5) without complications that would limit the subsequent use of blood products. For those in whom refractoriness was not documented, platelet transfusions were an effective method for controlling bleeding, or for performing surgical operations. It is important to note that in this study, refractoriness to platelet transfusion was determined based on clinical criteria since the measurement of autoantibodies is not part of the strategy in daily clinical practice, and access to diagnostic methods is limited for health institutions. However, indirect methods of platelet recovery are used in daily clinical practice [15].

**Figure 2:**
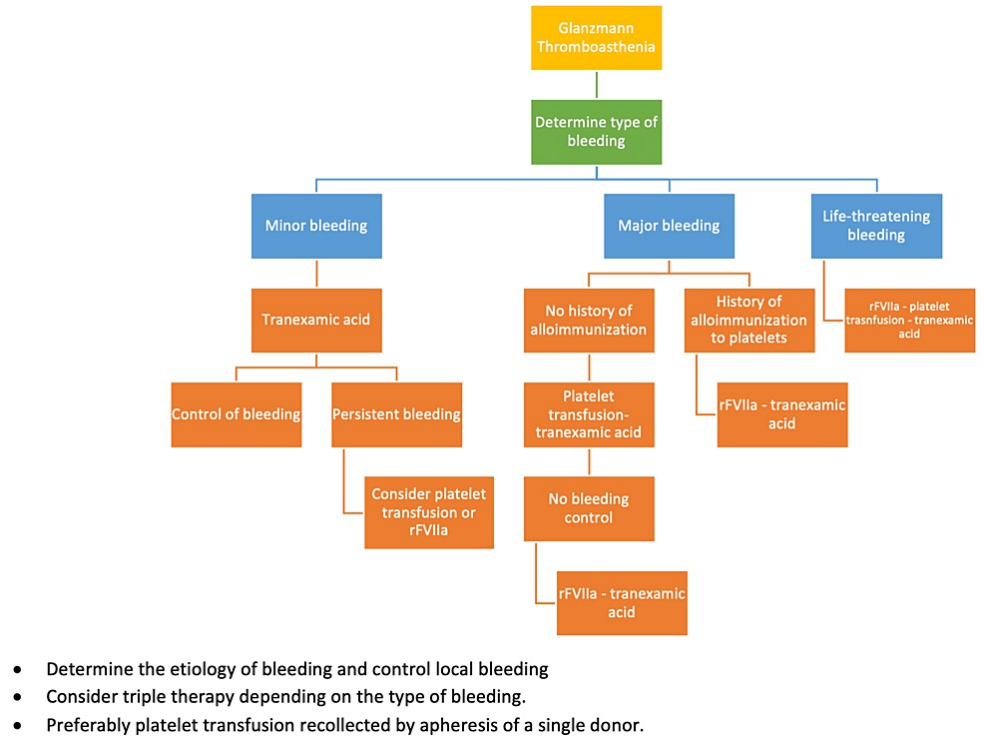
Proposed therapeutic algorithm to approach bleeding episodes in patients with Glanzmann thrombasthenia Tranexamic acid = TA; Platelet transfusion, recombinant Factor VIIa = rFVIIa

There are approved guidelines for the use of rFVIIa worldwide. In the country, it is indicated for the treatment of patients with Glanzmann's Thrombasthenia, for the treatment of hemorrhagic episodes, and for the prevention of bleeding in cases of surgery or invasive procedures when platelet refractoriness exists or when platelets are unavailable [22]. A proposed algorithm is depicted in Figure 2.

It was administered in 17 of the hospital admissions, as monotherapy in three cases, in combination with antifibrinolytics or platelets in seven, and as triple therapy in seven hospital admissions. No adverse events were reported during the administration period of the present study, and this is in line with what has been reported in world literature where venous thromboembolism is the main adverse event and has an incidence of <0.2% of treated patients [10, 23].

In this study rFVIIa alone or in combination was indicated for a similar number of patients of those who received platelet transfusions. There are differing reports in the literature, the oldest of which referred to a less frequent use of rFVIIa which was used preferentially as a rescue therapy. In the most recent reports, the use of rFVIIa is more frequent than platelet transfusions as first-line management to prevent bleeding in surgical situations [10, 15].

During the follow-up on the patients, three were taken to undergo repeat operations due to mechanical and platelet causes and one due to persistent bleeding, there was no mortality during hospital stays.

This study has some limitations. The first one is its retrospective nature since it covers a 10-year period of data collection during which therapeutic approaches could vary over time with possible changes in their indications. However, prospective randomized studies are hampered by the very low frequency of the disease. There are some prospective non-randomized reports in the literature reporting the clinical behavior in real life, and this is probably the best evidence available to date regarding this pathology. Furthermore, the registered platelet transfusion refractoriness was given by clinical criteria without antibody measurement, which is the recommended and standardized method for determining it.

## Conclusions

In conclusion, this is the most extensive registry of hospital admissions for patients with GT in Colombia and Latin America with descriptions of clinical characteristics, indications for hospitalization, and types of treatments. It must be recognized that GT is a difficult pathology to diagnose and is associated with significant bleeding episodes. It is also a challenge for hematologists and interdisciplinary teams with respect to the choice of treatments and related costs. Therefore, the early recognition and planning of strategies to prevent and treat bleeding are very useful for the patient and the health system.
